# A nationwide survey on the prevalence of asbestos-related lung cancer in Japan

**DOI:** 10.1371/journal.pone.0313468

**Published:** 2026-03-20

**Authors:** Mariko Niino, Taro Tomizuka, Yuka Nishina, Yuichi Ichinose, Wataru Gonoi, Takahiro Higashi

**Affiliations:** 1 Division of Health Services Research, Institute for Cancer Control, National Cancer Center, Chuo-ku, Tokyo, Japan; 2 Department of General Medicine, Juntendo University Faculty of Medicine, Tokyo, Japan; 3 Department of Public Health and Health Policy, Graduate School of Medicine, The University of Tokyo, Bunkyo-ku, Tokyo, Japan; 4 Departments of Radiology, The University of Tokyo, Bunkyo-ku, Tokyo, Japan; Sichuan University, CHINA

## Abstract

The nationwide prevalence of asbestos-related lung cancer (ARLC) needs to be accurately estimated to adequately operate a compensation subsidy program for patients with ARLC. In the present study, we aimed to estimate the proportion of patients with ARLC among patients with primary lung cancer according to the criteria established in the Japanese national compensation system, and described the characteristics and distribution of ARLC,. All facilities that treated patients diagnosed with lung cancer in 2016 were requested to submit computed tomography (CT) images of ten patients who were randomly selected from the national databases of hospital-based cancer registries. ARLC was defined as pleural plaques (PPs) extending over one-quarter of the inner lateral chest wall or existing PPs accompanied by obvious lung fibrosis. We estimated the proportion and distribution of ARLC among primary lung cancer cases and compared the characteristics of ARLC with those of primary lung cancer. Of the 772 facilities that treated at least one patient with lung cancer, 370 facilities provided 3,565 sets of CT images. Among them, 216 (6.1%) patients had PPs, and 86 (2.4%) patients met the compensation criteria. After sample weighting, 2.0% of all primary lung cancers were classified as ARLC in Japan. Compared with other primary lung cancers, a higher percentage of patients with ARLC were male (94.2% vs. 68.6%; *P* < 0.01) and had more advanced-stage disease (stage III: 22.1% vs. 16.0%; stage IV: 44.2% vs. 39.8%; *P* = 0.05). Most patients with ARLC (53.5%) were diagnosed at designated cancer hospitals. The proportion of patients with squamous cell carcinoma was higher in patients with ARLC than in those with primary lung cancer (25.6% vs. 18.6%; *P* < 0.01). The estimated number of patients with ARLC was higher than expected from the number of applicants in the Asbestos Health Damage Relief System for asbestos-related health damages. Thus, countermeasures are needed to accurately identify eligible compensation recipients.

## Introduction

Asbestos is classified as a group 1 carcinogen by the International Agency for Research on Cancer [1]. Because the time from asbestos exposure to the incidence of malignancy can span several decades, asbestos-related malignancies, either lung cancer or mesothelioma, are insidious [2,3]. In a previous Japanese study, the period from the first exposure to the appearance of asbestos-related lung cancer (ARLC) ranged from 5 years to 71 years, with a median of 47 years [4]. Meanwhile, a Swedish cohort study reported a mean latency period of >44 years [5]. Although the range of estimated incubation periods varies across surveys, the incidence rate of asbestos-related malignancies is expected to increase continuously in the coming years [6]. This expectation is based on the observation that the peak amount of imported asbestos rose during the 1980s [7]. Recent global estimates based on the Global Burden of Diseases Study 2021 indicate that Japan continues to bear a high mortality burden attributable to occupational asbestos exposure [8].

Although asbestos-related mesothelioma is rare, lung cancer accounts for the highest number of cancer deaths in men and the second-highest number of deaths in women in Japan [9]. Lung cancer has many known risk factors, including smoking [10] and air pollution [11]. In Asian settings, a hospital-based case–control study reported that cumulative asbestos exposure of ≥10 fiber-years was associated with an odds ratio exceeding 3 for lung cancer [12].However, the frequency of lung cancers caused by asbestos remains unknown, with a massive difference across studies [13,14], ranging from 4% to 12%. Although the government has initiated a rescue program for ARLC to compensate for the detrimental health effects related to the industrial use of asbestos, the number of applications remains small compare to the expected number of eligible individuals [14]. In addition to the regular workers’ compensation program, the Ministry of Environment’s Asbestos Health Damage Relief System (AHDRS) provides financial aid to patients solely on clinical findings, even those who are not covered by workers’ compensation programs because of insufficient evidence of the connection between their illness and work [15]. The subcommittee of the Central Environment Council in Japan, which oversees the operation of AHDRS, has also raised concerns that the number of applications appears smaller than the actual number of patients with ARLC [14].

The accurate prevalence of ARLC in the Japanese population is needed for the appropriate planning and operation of the compensation programs including AHDRS. However, previous studies have estimated the frequency of ARLC based on data from limited facilities specialized for occupational health diseases [7,16,17]. Thus, our study aims to accurately estimate the prevalence of ARLC among primary lung cancer nationwide and describe the characteristics of ARLC cases. Because the primary purpose of the study is to inform AHDRS with the case volume for compensation, we adopted the criteria of ARLC used in the AHDRS.

## Materials and methods

### ARLC criteria

To identify patients with ARLC, we used the criteria for computed tomography (CT) images established by the Asbestos Health Damage Assessment Subcommittee (AHDAS) of the Central Environment Council for the purpose of determining eligibility for compensation. Cases that met at least one of the following criteria were defined as ARLC: (1) pleural plaques (PPs) extending over one-quarter of the inner lateral chest wall and (2) presence of PPs accompanied by a clear shadow of pulmonary fibrosis indicating pneumoconiosis, with nodular or irregular opacity in both lung fields. The inner lateral chest wall line was defined as a curve that runs ventrally from the sternal border and dorsally to the origin of the ribs. For multiple plaques (including those observed in the mediastinal pleura in the same section), the total length of the plaques was measured.

The justification of the criteria is as follows: According to the Helsinki Consensus, these criteria are considered equivalent to a cumulative exposure of 25 fiber-years [18]. A twofold increased risk of lung cancer is associated with retained fiber levels of 5 million amphibole fibers (>1 μm) per gram of dry lung tissue. This lung fiber burden is approximately equal to 5,000–15,000 asbestos bodies, comprising asbestos fibers coated by iron-containing protein and mucopolysaccharide per gram of dry lung tissue [19]. PPs are caused by the inhalation of fibrous silicate minerals, including asbestos or erionite. Yusa et al. [20] reported that among the cases wherein >5,000 asbestos bodies per gram of dry tissue were detected, 75% of cases showed that the extent of PPs was > 25% of the inner chest wall.

### Subjects

The target population included patients with primary lung cancer diagnosed from January to December 2016. The cases were obtained from a hospital-based cancer registry (HBCR) database bearing the codes indicating invasive cancer (behavioral code 3) of the lung (C340-C349 by ICD-O-3 topography code) for patients who underwent initial treatment at the registering hospital.

### HBCRs and cancer care hospitals

The operation of an HBCR is mandated as a condition for designation as a cancer care hospital. These hospitals were designated by the Ministry of Health, Labour and Welfare to specifically provide cancer care in their respective communities or local regions. Some nondesignated hospitals that play central roles in their localities also maintain an HBCR. The designated and nondesignated hospitals submit data to the National Cancer Center annually, the central database of which is estimated to cover approximately 70% of new cancer cases in Japan [21]. The personal identifiers in the HBCR are removed and replaced with identifying labels assigned solely for tracing. Each medical facility maintains a catalog linking the identifying labels and personal identifiers.

We requested to use data from 772 medical facilities that submitted lung cancer cases in 2016 to the National Cancer Center. Among these cases, we randomly selected ten patients from each facility and requested the participating hospitals to obtain their chest CT images. If fewer than ten lung cancer cases were treated at a facility in 2016, all registered lung cancer cases were requested for enrollment in the survey. Moreover, we collected CT data taken nearest the date of lung cancer diagnosis into an electronic file.

### Evaluation of CT images

The CT images were evaluated in two phases. In the first phase, 12 pulmonologists and 12 radiologists identified suspected ARLC cases. These first-phase readers checked for PPs. If PPs are present, the readers documented the extension of PPs, presence of calcification, anatomical location of PPs, and presence of pulmonary fibrosis. Two readers independently analyzed every CT image. If the readings of the two readers differed, a third reader analyzed the relevant CT images. In the second phase, five radiologists specialized in identifying ARLC analyzed the CT images. These radiologists all had experience in serving on the approval board of AHDRS as a member of the AHDAS. They reviewed suspected cases that were identified by at least one reader during the first phase. Difficult cases that the experts did not want to judge alone were further discussed at a review meeting. We prioritized the experts’ opinions on the images that had disagreements in judgment between the first and second phases.

### Statistical analysis

The percentages of patients with PPs and those that satisfied the ARLC criteria among all sampled patients with lung cancer were calculated. The percentages of patients with ARLC were weighted against the total number of patients with lung cancer for each enrolled facility to estimate the proportion of patients with ARLC among those with primary lung cancer. The weights in each facility were calculated by dividing the number of registered patients with primary lung cancer by the number of samples from each facility.

To determine the characteristics of patients with ARLC, we compared the ARLC group with the non-ARLC group (patients with other types of lung cancer) with respect to the following parameters: sex, age, geographic region, type of diagnostic facility, primary lung cancer site, histological type, and cancer stage. Chi-square tests and Student’s t-test were performed to compare proportions and means between the two groups, respectively. All tests were two-sided, and statistical significance was considered at *P* < 0.05. To determine the distribution of patients with ARLC according to the type of medical facility, we categorized the facility where the patients were diagnosed with lung cancer as a designated cancer hospital, laborers’ hospital, or another type of hospital by reviewing the subject’s HBCR data and then compared the proportions. Moreover, we compared the differences in CT image readings between generalists and asbestosis specialists to obtain insights on the difficulty of diagnosis.

All statistical analyses were performed using Stata 15 software (StataCorp LLC, College Station, TX, USA). This study was approved by the Institutional Review Board of the National Cancer Center, Japan (2018−193). The survey was performed from October 2018 to March 2020. During the two-year review period, we accessed the collected anonymized chest imaging data. Given that imaging data were anonymized, we did not have access to information that could identify individual participants during or after data collection. We facilitated the disclosure of research information and accommodated opt-outs.

## Results

### Estimated proportion of ARLC in primary lung cancer

**Fig 1:**
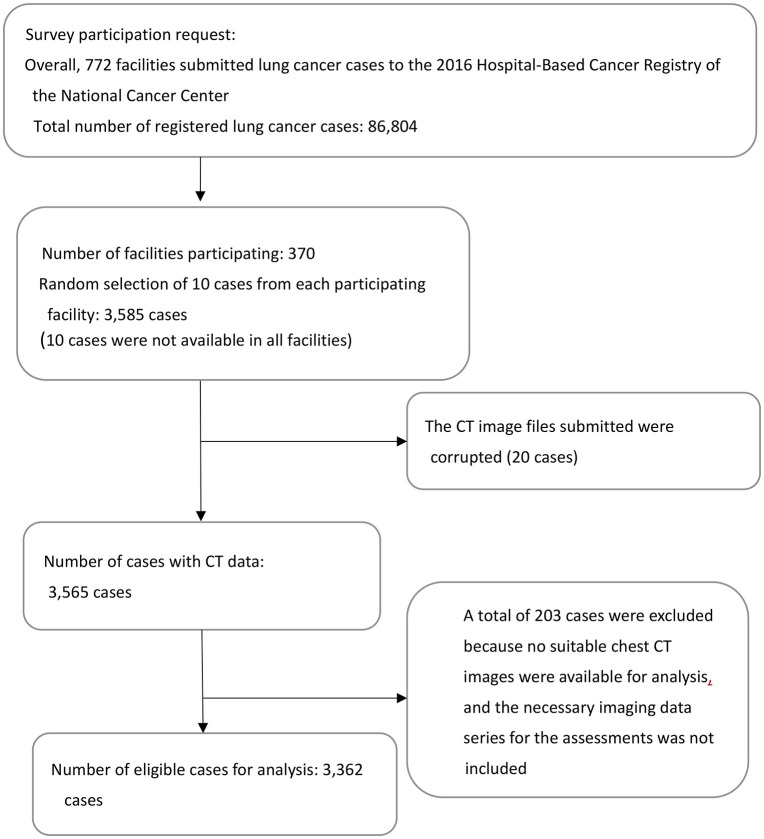
Flow chart of patient inclusion in the survey.

The number of CT images that were eligible for analysis is shown in Fig 1. In this survey, 370 facilities were enrolled, and the CT series of 3,585 patients was collected. The image data from 20 patients were excluded because the files that were submitted were corrupted. Thus, data on 3,565 patients were used for analysis. The CT images from 203 patients were excluded because no suitable chest CT images were available for analysis and the necessary imaging data series for the assessments was not included. Finally, a total of 3,362 cases were available for analysis. Table 1 shows the characteristics of the selected patients compared with those of all patients with primary lung cancer in the 2016 HBCR (n = 86,804). Regarding the distribution of histological subtypes, adenocarcinoma accounted for 41.5%(n = 967) of male patients and 68.3%(n = 706) of female patients. This sex-specific pattern is consistent with previous reports in Asian populations, where adenocarcinoma is more prevalent among females.

**Table 1:** Characteristics of patients with primary lung cancer in the HBCR.

Category		2016 HBCR		Patients for analysis	
		(n = 86,804)	(%)	(n = 3,565)	(%)
Sex	Male	58,492	67.4	2,476	69.5
	Female	28,312	32.6	1,089	30.6
Age at diagnosis, years	Under 39	477	0.6	13	0.4
	40–49	2,049	2.4	69	1.9
	50–59	6,460	7.4	224	6.3
	60–69	25,918	29.9	955	26.8
	70–79	33,643	38.8	1,308	36.7
	80–89	16,729	19.3	879	24.7
	Over 90	1,528	1.8	117	3.3
Histological type	Small-cell carcinoma	7,326	8.4	327	9.2
	Squamous cell carcinoma	16,701	19.2	668	18.7
	Adenocarcinoma	47,547	54.8	1,758	49.3
	Others	15,230	17.6	812	22.8
Primary site of lung cancer	Bronchus	3,207	3.7	183	5.1
	Upper lobe	43,609	50.2	1,747	49
	Middle lobe	4,724	5.4	168	4.7
	Lower lobe	33,512	38.6	1,360	38.2
	Border	56	0.1	4	0.1
	NOS	1,696	2	103	2.9

HBCR, hospital-based cancer registry; NOS, not otherwise specified

**Fig 2:**
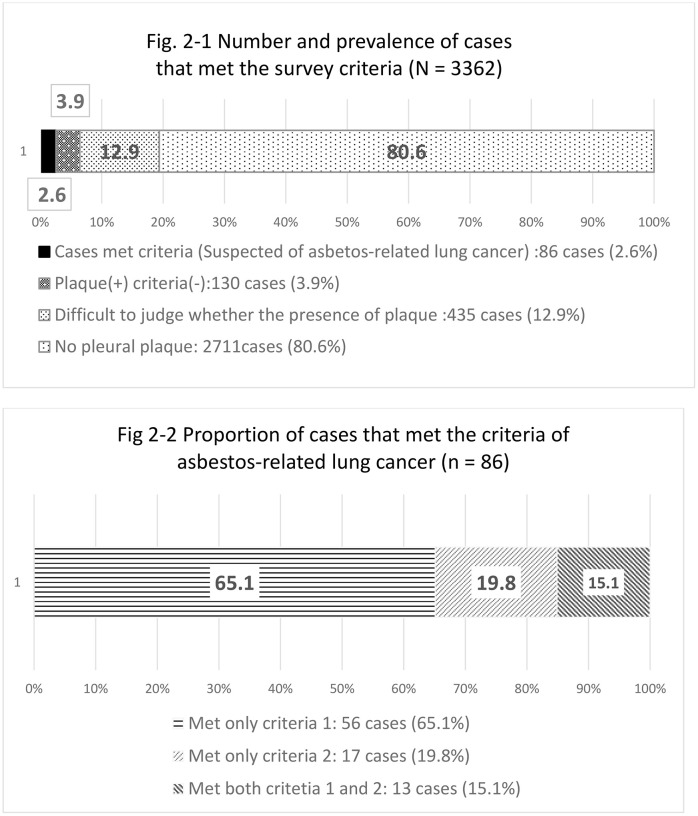
Number and prevalence of cases that met the survey criteria.

Among the 3,362 patients, 216 patients (6.4%, 95% confidence interval [CI] 5.6%–7.3%) were diagnosed with PPs, and 86 patients (2.6%, 95% CI 2.1%–3.1%) met the criteria for ARLC. In 12.9% of cases, the presence of PPs could not be determined from the CT images. These cases were classified as not meeting the criteria for ARLC because no clear fibrosis was identified. PPs were not detected in 80.6% of patients with lung cancer (95% CI 79.3%–81.9%) (Fig 2−1). Among the 86 patients with PPs, 69 patients (80.2%, 95% CI 70.3%–87.4%) had PPs extending over one-quarter, whereas 17 patients (19.8%,95% CI 12.6%–29.9%) had PPs with obvious pulmonary fibrosis. After applying weightings, 2.0% of patients with primary lung cancer registered in the 2016 HBCR (95% CI 1.5%–2.6%) were estimated to have ARLC (Fig 2−2).

In the second phase of CT evaluation conducted by radiology specialists, 26 patients demonstrated a clear shadow of pulmonary fibrosis indicating pneumoconiosis, with nodular or irregular opacities, but without detectable or clearly identifiable pleural plaques. According to the predefined study criteria, these cases were not classified as asbestos-related lung cancer (ARLC) and were therefore excluded from the ARLC count.

### Differences in criteria judgment between physicians and radiological experts for ARLC

The radiologists in the second phase with experience in assessing AHDRS compensation recipients using the ARLC criteria reviewed 503 cases. At least one reader suspected PP extension or was satisfied with the ARLC criteria on CT images determined during the first reading phase, including 134 cases judged as meeting the ARLC criteria. The physicians and radiologists in the first phase and the radiological experts on ARLC differed in their assessments on 86 cases. Among these cases, 67 cases were rejected by the radiological experts, and 19 cases that were rejected by the first-phase physicians were judged as meeting the criteria for pulmonary fibrosis or PP extension by the experts. The main reason for rejection by the experts (41 cases) was misreading of PPs on CT by the physicians.

### Demographic and histological characteristics of patients with ARLC

The characteristics of patients with and without ARLC are shown in Table 2. The ARLC group had more males than the non-ARLC group (94.2% vs. 68.6%; *P* < 0.01). The age at diagnosis tended to be higher for patients with ARLC than for those with other types of lung cancer (71.7 vs. 68.2 years; *P* < 0.01). The proportion of squamous cells was significantly higher in the ARLC group than in the non-ARLC group (25.6% vs. 18.6%; *P* < 0.01) (Table 2). ARLC cases were identified in facilities across all regions of Japan. The proportion appeared slightly higher in facilities located in industrialized areas such as the Kinki region (15.7% vs. 20.9%, n = 18), the Chugoku region (8.7% vs. 16.3%, n = 14), and the Kyushu/Okinawa region (12.6% vs. 21.0%, n = 18), whereas lower proportions were observed in facilities in the Hokuriku region (7.7% vs. 1.2%, n = 1), the Tokai region (9.6% vs. 4.7%, n = 4), and the Kanto region (19.2% vs. 11.6%, n = 10).

**Table 2:** Comparison of the demographic and histological characteristics of patients with ARLC and those with other lung cancers.

Characteristic/demographic factors		Survey criteria				
	Categories	ARLC^*1^ (−)		ARLC (+)		
		(n = 3,276)	(%)	(n = 86)	(%)	*P*-value
Sex	Male	2,248	68.62	81	94.19	<.01^*2^
	Female	1,028	31.38	5	5.81	
Age at diagnosis, years	Under 39	12	0.4	0	0	
	40–49	67	2.1	1	1.2	
	50–59	213	6.5	0	0	
	60–69	878	26.8	17	198	
	70–79	1,207	36.8	39	45.4	
	80–89	796	24.3	23	26.7	
	Over 90	103	3.1	6	7	
	Average age (SD*^6^)	68.2	(11.1)	71.7	(9.1)	<.01^*3^
Primary site	Bronchus	172	5.3	4	4.7	.62*^4^
	Upper lobe	1,613	49.2	38	44.2	
	Middle lobe	157	4.8	2	2.3	
	Lower lobe	1,238	37.8	39	45.3	
	Border	4	0.1	0	0	
	NOS^*5^	92	2.8	3	3.5	
Stage	NOS	16	0.5	0	0	.05*^4^
	Ⅰ	1,070	32.7	16	18.6	
	Ⅱ	253	7.7	11	12.8	
	III	523	16	19	22.1	
	Ⅳ	1,305	39.8	38	44.2	
	Unknown	109	3.3	2	2.3	
Histological type	Small-cell carcinoma	299	9.1	11	12.8	<.01*^4^
	Squamous cell carcinoma	608	18.6	22	25.6	
	Adenocarcinoma	1,648	50.3	25	29.1	
	Others	721	22	28	32.6	

*1: ARLC, asbestos-related lung cancer

*2: Chi-square test

*3: *t*-test

*4: Fisher’s exact test

*5: NOS, not otherwise specified

*6: SD, standard deviation

### Distribution of ARLC cases by type of medical facility

The percentage of patients with ARLC among patients with lung cancer at the labor accident hospital (Rosai Hospital) was higher than those of other hospitals (8.2% vs. 2.4%; *P* < 0.01). However, 53.5% of patients with ARLC were diagnosed at a designated cancer hospital, whereas 37.2% were diagnosed at other medical facilities.

## Discussion

Our study showed that 2% of patients with lung cancer met the ARLC criteria. Based on the annual incidence of lung cancer of approximately 80,000 cases [22], we expect 1,600 patients to be diagnosed with ARLC annually, which is much larger than the total number of applications made to AHDRS and for workers’ compensation insurance. In 2016, 431 and 134 people were compensated by workers’ compensation and AHDRS, respectively [23,24]. This finding might suggest that a significant number of patients have been inaccurately diagnosed with ARLC. The lack of awareness among healthcare professionals regarding the AHDRS for application for ARLC compensation might also contribute to discrepancies between the estimated numbers of our results and the actual number of compensation recipients. Moreover, the estimated percentage of ARLCs highlights the number of potential patients who have not applied for asbestos compensation relief. Similarly, studies in other countries have reported that barriers to rescue systems for asbestos victims are related to underreporting [25]. The lack of awareness of reportable conditions [26] and time constraints associated with completing multiple record requirements and involvement in government or legal hassles can contribute to underreporting by physicians [27]. Moreover, physicians tend to underreport ARLC, especially if the patient is a smoker [28].

The radiological imaging criteria above indicate that the patient had had cumulative asbestos exposure, increasing the risk of lung cancer by twofold [29]. These criteria are useful because, although there is a correlation between cumulative asbestos exposure and lung cancer risk [30], accurately determining the level of exposure solely through exposure history interviews is challenging because of its reliance on the patient’s memories. In Japan, under the AHDRS, lung cancer is regarded as asbestos-related when the above criteria are met. Although ARLC diagnosis is made clinically taking into account many factors, this study employed the AHDRS criteria operationally

In previous studies in Japan that estimated the percentage of ARLC cases, the study sites included medical facilities specialized for work-related diseases. Therefore, their estimates were expected to be higher than those in a general population [16]. The present study included nonspecialized medical facilities, which makes our results more generalizable than those of previous studies. Furthermore, we found that a large proportion of patients with ARLC were diagnosed at regular designated cancer care hospitals, not at facilities specializing in work-related diseases. If we are to evaluate the incidence of ARLC appropriately, we should include nonspecialized hospitals.

Notably, 17% of diagnoses (86/503 cases) differed between physicians in the first phase and radiological experts for ARLC in the second phase. A comparison of diagnoses between the results of the first and second phases revealed that non-experts more frequently made false-positive diagnoses. The discrepancies were mainly because of differences in determining the presence or absence of PPs. There are several possible reasons for these discrepancies. First, general physicians have few opportunities to compare and discuss the interpretation of PPs on chest CT scans in clinical practice because PPs are not usually related to lung cancer treatments. Second, the pleural thickness and intercostal veins of pulmonary lesions caused by chest wall contact present characteristics similar to those of PPs, making them difficult to distinguish. Moreover, PPs are sometimes difficult to distinguish from false-positive findings on radiological images. The survey results indicate that a certain number of cases are rejected during AHDRS accreditation by physicians who submit false-positive findings. Because of substantial paperwork, a false-positive finding would waste the time of physicians and patients. Further continuing education on the accurate diagnosis of PPs before submitting applications to the AHDRS is needed. In addition, the potential strategies include promoting application submissions even with false positives to minimize omissions and exploring the development of automated detection systems.

We found a higher proportion of squamous cell carcinomas in ARLC than in non-ARLC cases (35.6% vs. 18.6%). Our results showed that the proportion of squamous cell carcinomas was relatively higher in ARLC cases than in non-ARLC cases, which might have been influenced by the higher proportion of smokers in the asbestos-related disease cohort than in the general population because smoking synergistically increases the risk of asbestos-related lung disease, and the said cohort has a high risk of developing squamous cell carcinoma [31].

A comparison of cancer stages at the time of diagnosis showed that ARLC tended to be more advanced on diagnosis compared with other lung cancers (stage III: 22.1% vs. 16.0%; stage IV: 44.2% vs. 39.8%). Generally, the prognosis of ARLC is poor because the cancer develops in older patients due to the long incubation period and low lung function attributed to lung fibrosis and emphysema.

### Limitations

Our study has several limitations. First, we only analyzed CT images. The response rate was 55%, which is reasonable because we conducted a national survey. However, the number may not be representative of the population with lung cancer in Japan. Despite this, we included as wide a variety of hospitals as possible and implemented a random sampling design. Second, the determination of ARLC applied compensation criteria based on the presence of PPs of a specified length, which is associated with pulmonary asbestos body counts that increase the risk of lung cancer by twofold. These criteria do not cover all the factors and findings that have been epidemiologically identified about asbestos and lung cancers. Other findings, including the types of asbestos exposed, length of exposure, and nodules or irregular opacity, may indicate ARLC. However, in this study we focused on pleural plaques (PPs) as the case definition, because the primary aim was to estimate the number of cases who can be compensated by the AHSRS. In this study, some lung cancer cases showed radiological findings consistent with a clear shadow of fibrosis indicating pneumoconiosis with nodular or irregular opacity in the absence of pleural plaques. Although these findings were not included in the predefined criteria for ARLC, such cases may still represent lung cancers potentially attributable to asbestos exposure. Third, the incidence of ARLC may be changing chronologically. We surveyed patients diagnosed in 2016, indicating that repeated studies are warranted to show any potential trend over time. Fourth, we only collected a limited amount of background information on the patients. Occupational and smoking history data would have broadened the analysis from our data. However, given the difficulty in obtaining these data, asking for these data may have decreased the response rate of the hospitals. We placed a higher priority on recruiting hospitals to estimate the prevalence of ARLC as accurately as possible and did not collect information exhaustively to avoid burdening the hospitals. Fifth, we did not have the resources to re-evaluate missed findings from Phase one. However, a double-check process was implemented in the initial phase, and cases with uncertain findings were included in Phase two. Finally, to simplify the data collection process, we did not collect simple chest radiographs and judged fibrosis based on the CT images, which may have overestimated the incidence of the second criterion because CT is much more sensitive than chest X-ray imaging in detecting fibrosis. However, the potential overestimation would not considerably change our results because >80% of the patients met the criteria of having widespread PPs.
